# Volume Transmission and Pain Perception

**DOI:** 10.1100/tsw.2003.53

**Published:** 2003-08-02

**Authors:** Gilberto C. Castañeda-Hernàndez, Paul Bach-y-Rita

**Affiliations:** ^1^ Centro de Investigación y Estudios Avanzados del Instituto Politécnico Nacional, Avenida Instituto Politécnico Nacional 2508, México, D.F. 07300, Mexico; ^2^ Departments of Orthopedics and Rehabilitation Medicine, and Biomedical Engineering, University of Wisconsin-Madison, 1300 University Avenue, Madison, Wisconsin, USA

**Keywords:** volume transmission, pain perception, nonsynaptic diffusion neurotransmission, diffusion, brain energetics, neuronal function, glial function

## Abstract

Volume transmission (VT) is the diffusion through the brain extracellular fluid of neurotransmitters released at points that may be remote from the target cells with the resulting activation of extrasynaptic receptors. VT appears to play multiple roles in the brain in normal and abnormal activity, brain plasticity and drug actions. The relevance of VT to pain perception has been explored in this review.

## INTRODUCTION

Volume transmission (VT) includes the diffusion through the extracellular fluid of neurotransmitters released at points that may be remote from the target cells, with the resulting activation of extrasynaptic receptors (and possible intrasynaptic receptors reached by diffusion into the synaptic cleft). VT also includes the diffusion of substances such as nitric oxide (NO) and carbon monoxide (CO) through both the extracellular fluid and cellular membranes. In contrast to the one-to-one, point-to-point "private" intercelleular synaptic communication in the brain, VT is a slow, one-to-many, widespread intercellular communication[1]. VT appears to play multiple roles in the brain in normal and abnormal activity, brain plasticity, and drug actions (reviewed[2,3,4,5,6]); in some reactions, such as those experienced with psychotropic drigs VT mechanisms may predominate[7]. Combinations of both synaptic and diffusion neurotransmission are important in vision and many other functions.

While the synaptic basis of information flow (also called the Cajal-Sherington paradigm of interneuronal communication) has been the major focus of neuroscience research for more than a century, experimental results obtained in VT studies have led various scientists, including Agnati et al.[3], to consider that it is time to revise that paradigm. They noted that although much work remains to be accomplished to firmly establish the concept, VT must now be considered to play an important role in neuronal communication. One of the authors (PB) has previously used the term NDN (nonsynaptic diffusion neurotransmission) instead of VT. VT has clearly been the term preferred by investigators, and thus will be used here.

Included among the many functions in which VT plays a role are those mediated by noradrenaline: in the autonomic nervous system (e.g.,[8]), in human omental veins[9], and in the central nervous system (c.f.[10])VT may also play a role in functional reorganization following brain damage[5]. VT appears to be the principal means of neurocommunication in many invertebrates (c.f.[5,11]), and may be more common in certain mammalian brain regions. VT may be the primary information transmission mechanism in certain normal mass, sustained functions, such as sleep, vigilance, hunger, brain tone and mood[12], and certain responses to sensory stimuli, as well as several abnormal functions, such as mood disorders, spinal shock, spasticity, shoulder-hand and autonomic dysreflexia syndromes, and drug addiction[5,13,14].

Work carried out in Routtenberg's laboratory in the late 1960s (reviewed by him in 1991[15]) suggested that transmitters could readily move in the extracellular space (the fluid that surrounds neurons in the brain) and travel over distances by directional flow. This has been confirmed and extended by Bjelke et al.[16]. Furthermore, a large number of receptor subtypes have been identfied; these provide a mechanism for selective activation even if the VT neurotransmitter is massively diffused over an area. High affinity receptors on cells distant from the transmitter release can bind the neurotransmitter and cause a neuron response, while closer neurons without those specific receptor subtypes will not respond[17].

VT may be more common in certain brain regions; nonsynaptic interneuronal communication is very common in the greater limbic system[18] and may play an important role in the organization and regulation of behavior by the core and paracore regions of the brain[11]. VT may also be an important mechanism in the highest levels of the human brain. Physical anthropologists are puzzled by the fact that the human brain does not use more energy than the smaller brains of animals of comparable corporal weight; Bach-y-Rita and Aiello have suggested that the parts of the human brain that show the greatest size increase over other animals, such as prefrontal cortex, may be exactly those parts in which highly nonsynaptic-based functions have their neuronal representation. Music appreciation and intellectual functions, for example, may not require the high-frequency (and energetically costly) alternating cycles of activation-inactivation of synaptic transmission, and may be largely replaced by VT[17]. VT may play a role in recovery from brain damage, which causes changes in neurotransmitter levels. Some neurotransmitter systems are up-regulated, while others are down-regulated[19]. Drug therapy and rehabilitation may induce functional recovery by influencing the affected neurotransmitter systems.

In the 1950s, in order to study the action of GABA analogues on thalamic mechanisms, Killam and his colleagues injected them into cat ventricles, since they did not cross the blood-brain barrier[20,21]. At the time, we did not consider diffusion neurotransmission as a mechanism of action. However, later intra- and extracellular microelectrode studies in cat brainstem resulted in the suggestion that diffusion neurotransmission was the mechanism, and that it could play a role in the multiplexing of the polysensory cells[22]. This led to a proposed law of the conservation of space and energy in the brain[23]. These conceptual issues have been discussed elsewhere[5,24,25].

In 1968, Fuxe and his collaborators[26] began a series of immunohistochemical studies that led to highly productive VT studies (reviewed in[2,3]). Beaudet and Descarries[27] showed in 1978 that the biogenic amines released from nonsynaptic varicosities may act not only upon adjacent postsynaptic surfaces, but also in tissue of more distant receptor elements. Descarries has repeatedly confirmed and extended his earlier findings (c.f.[3]). Intrinsic and relational features of the monoamine and acetylcholine innervations are comparable during their early postnatal development to what they described in the adult, including their largely asynaptic character, which is apparent from the very beginning. This is now clear from a recent report [28] of the acetylcholine innervation in the developing parietal cortex of rat.

Neurochemical transmission by extrasynaptic diffusion had also been noted by Vizi, whose studies on the nonsynaptic modulation of transmitter release[29,30] led him to propose[31] that the essence of brain function (e.g., learning, thinking) lies not in variations of neuronal circuitry (hardware) but rather within the chemical communication itself which is partly wired (synaptic) and partly unwired (nonsynaptic) transmission.

The important role of glia in neuronal communication has been increasingly recognized in recent years. Syková and her associates (c.f. [3]) have shown how VT provides a conceptual framework for understanding how glia can participate in neurotransmission in the absence of nerve fibers connecting glia to neurons. Syková and Chvátal[32] also noted that during activation, neurons release not only neurotransmitters, but also a variety of other neuroactive substances such as ions that diffuse through the extracellular fluid to other cells.

How information is carried from one part to another in the brain has a great influence on the brain's space and energy. VT may provide a low-metabolic-cost alternative to synaptic communication. The cost of an action potential has been calculated[33] to be about $10^{11}$ to $10^{12}$ ATP per $cm^2$ of depolarized membrane, with a minimum cost of $10^6$ ATP at a node of Ranvier. Hebb[34] envisioned fully synaptically connected cell–assemblies to explain mental states, such as thought, expectancy, interest and attention. However, such connectivity would require so much energy that it might exceed metabolic resources; a small (10,000 neuron) module would require approximately 1 J/l of brain. Furthermore, the volume of the nerve fibers necessary to fully connect the brain synaptically would create a brain so large that it would not fit in the head [35]. Rather than purely synaptic or purely diffusive, it is likely that hybrid neuronal networks, exhibiting varying combinations of hard-wired (synaptic) and wireless (VT) connectivities, would fit into the available volume in the brain, with a significant cut in space and energetic costs resulting from removing a significant number of "hard" links (and the nerve fibers needed to connect them) and replacing them with "soft", diffusive pathways[36].

## THE RELEVANCE OF VT FOR PAIN PERCEPTION

Our comprehension of the mechanisms involved in pain perception (nocioception) has advanced greatly in the last decades. It is now clear that pain is generated and modulated at peripheral, spinal and supraspinal sites[37]. Moreover, although a tissue injury generates a painful stimulus, the intensity of the resulting pain is not necessarily proportional to the extent of the injury. This is due to the fact that pain perception is modulated by a series of biological and psychological factors that we are now starting to understand[37,38]. However, there are many aspects of the neurophysiological basis of pain that remain obscure. Several theories have been proposed to explain nociception, but none of them can satisfactorily describe all aspects of pain[39]. It is generally assumed the nociceptive signal generated after a noxious stimulus is transmitted by synaptic mechanisms[40,41]. However, there are data pointing that VT is involved in the generation and modulation of pain[5,42]; the evidence is reviewed below.

### Processes of Nociception

Nociception is considerably more complicated than a simple detection of tissue injury. A tissue can be injured by several factors such as heat, pressure, radiation, and chemical agents. Nociceptors, or pain receptors, can extract information from all these sources of energy. This information is then transformed into electrical stimuli, i.e., action potentials, in a peripheral neuron and then processed in the central nervous system. In this way, nociception is constituted by four processes [43]:

1. *Transduction* is the transformation of noxious stimuli causing tissue injury to electrical signals in the peripheral neurons.
2. *Transmission* is the propagation of electrical signals along nociceptive nerve fibers.
3. *Modulation* is the alteration (amplification or attenuation) of nociceptive signals in the spinal cord and at supraspinal sites.
4. *Perception* is the process of the integration of nociceptive signals arriving to the brain with cognitive and emotional factors, yielding the subjective experience known as pain.

After a tissue injury, a variety of mediators are released either by the injured tissue or by macrophage and other cells arriving at the site of injury as part of the defense mechanisms triggered by the organism. Among these mediators are bradikinin, substance P, histamine, prostaglandins, nitric oxide, protons, ATP, serotonin, excitatory aminoacids, kinins, and many others[37,44,45]. These mediators diffuse through the intracellular fluid arriving to the primary neuron, i.e., the one located near the injured tissue. The primary neuron is thus activated, sodium channels are opened, and action potentials are generated[37]; that is, the transduction process takes place. Moreover, other mediators can be released by other neurons located in the vicinity, which can also diffuse and reach the primary neuron, modulating the transduction process[44]. It is therefore clear that transduction includes a nonsynaptic process of neuron activation.

After a noxious stimulus, action potentials are conducted mainly by C-fibers, although Aα-, Aβ-, and Aδ-fibers can also be involved, arriving at the dorsal horn of the spinal cord where the nociceptive signal is modulated[37,46]. Most neurons in the dorsal horn have a receptive field, i.e., each neuron receives information from a particular body region that stimulation increases neuronal activity. Thus, receptive fields appear to be arranged as a somatotopic map. This arrangement, however, is far more complex, as receptive fields exhibit considerable overlapping. Moreover, there is a remarkable degree of convergence, as afferent fibers from several body regions can be projected to a single spinal neuron in the dorsal horn. Furthermore, it is now well known that excitatory and inhibitory interneurons also participate in the modulation of the nociceptive signal in the spinal cord[46].

Many mediators are involved in the modulation of the nociceptive signal at the spinal level, some of them excitatory and others inhibitory[41,46]. Among the mediators that have been described are excitatory aminoacids, prostaglandins, monoamines, nitric oxide, endorphins, GABA, adenosine, substance P, and neuropeptide Y. Due to the large number of neurotransmitters that have been identified as participants in the processing of the nociceptive signal in the spinal cord, as well as the complicated arrangement of neurons in the dorsal horn, it seems improbable that spinal modulation of nociceptive signals at the spinal cord occurs exclusively by synaptic mechanisms. It appears reasonable to postulate that VT can also be involved in such process. Information needing a high degree of precision, such as the localization of the anatomical site of injury, is very likely processed by synaptic mechanisms. On the other hand, other processes, such as the amplification and attenuation of the nociceptive signal, can likely be achieved with a sufficient degree of efficiency by VT with a considerable economy of energy[36]. The participation of nonsynaptic mechanisms in pain modulation has previously been suggested by other authors. Carlton and colleagues[47] have described a nonsynaptic mechanism of glutamate-induced release of noraderenaline in postganglionic sympathetic axons which is probably involved in pain states with a sympathetic component. Ridet and collegues[48] suggested that the monoaminergic systems involved in pain modulation in the dorsal horn of the spinal cord act partly through nonsynaptic diffusion transmission, as dopaminergic, noradrenergic, and serotonergic projections exhibit a nonsynaptic organization.

The modulated nociceptive signal is transported from the spinal cord to supraspinal sites by monosynaptic and polysynaptic pathways. The neuroanatomy and organization of the supraspinal sites is extremely complex. In fact, the actual mechanisms of pain perception have not been fully elucidated at present, although it is clear that emotional and learning processes have a significant influence on the overall experience of pain in a given individual[38,49]. As in the spinal cord, an important number of neurotransmitters are involved in nociceptive transmission in the brain. Glutamate, noradrenaline, and substance P are among the excitatory mediators, while dinorfin and GABA, which are inhibitory mediators, have also been identified in the brain sites involved in the processing of nociceptive information. There is evidence suggesting that VT plays a relevant role in nociception at the supraspinal level. Nothias and coworkers[50] have described the presence of noradrenergic and serotonergic fibers in the ventrobasal complex of the rat thalamus (VB), a structure known to be involved in nociception. Synaptic differentiation was not observed at the level of apposition of membranes between monoaminergic afferents and VB neuronal profiles. These observations thus suggest a nonsynaptic modulation of VB neuronal activity by monoaminergic afferents. Taylor[51] has reported that the analgesic effect of gabapentin involves nonsynaptic actions of GABA, suggesting that VT plays a role in the attenuation of the nociceptive signal by inhibitory neurotransmitters in the brain. It is hence likely that, in the brain, as in the spinal cord, the processing of nociceptive information is the result of both synaptic and nonsynaptic mechanisms.

## Secondary Hyperalgesia and Nonsynaptic Transmission

When there is a noxious stimulus, pain frequently is associated to two different zones. A first zone of primary hyperalgesia represents the injured tissue and is characterized by spontaneous pain and an increased sensitivity. A zone of secondary hyperalgesia also gradually develops, involving the region in the vicinity of the injured tissue [46,52]. Primary hyperalgesia can be satisfactorily explained by peripheral and central mechanisms involving synaptic transmission, including information on the precise anatomical localization of the injured site. However, secondary hyperalgesia cannot be explained by such a mechanism[46]. VT thus appears as an attractive explanation for secondary hyperalgesia. The primary nociceptive signal, i.e., that directly related to tissue injury, arrives at the spinal cord by a synaptic mechanism. In the dorsal horn, the nociceptive signal triggers the release of excitatory neurotransmitters that amplify the nociceptive signal. These excitatory neurotransmitters, however, act through a nonsynaptic mechanism, being released in the extracellular fluid, reaching other neurons in the vicinity, and thus reducing the threshold to trigger a nociceptive response in those sites innervated by these neurons. Experimental observations on the role of nonsynaptic mechanisms in spinal wind-up are consistent with our hypothesis. Wind-up is a gradual increase of the neuronal response to a constant stimulus. Although wind-up is not equivalent to secondary hyperalgesia, it shares some common mechanisms with this phenomenon[52]. Morisset and Nagy[53] described a nonsynaptic component of wind-up in dorsal horn spinal neurons.

VT likely participates in the modulation of the nociceptive signals at the spinal level. The signal is amplified or attenuated, depending on the overall influence of excitatory and inhibitory neurotransmitters present in the milieu of the neurons located in the dorsal horn. This is analogous to the situation of the primary neuron at the peripheral level. If the primary neuron is exposed to excitatory mediators, such prostaglandins or glutamate, the nociceptive signal is amplified[44,45]. If the primary neuron is exposed to inhibitory mediators, such as nitric oxide or endorphins, the nociceptive signal is attenuated[44,54]. There is no doubt of the role of VT in the transduction processes. VT may also play a role in the modulation of nociceptive signals at spinal and supraspinal sites, and in visceral pain in which the localization of pain is not precise.
